# Resorcin Poisoning in an Infant

**Published:** 1902-02

**Authors:** 


### Resorcin Poisoning in an Infant.

Dr. A. Caillé, in a recent number of *Pediatrics,* reports an interesting case of resorcin poisoning, the patient being an infant five days old. In the treatment of an ordinary dyspeptic diarrhea the child had been given one-fourth grain of resorcin every four hours, and after the sixth dose it became cold, clammy, cyanotic and pulseless and its urine smoky. By way of treatment a hot bath (110° F.) was given every two hours, the bowels were flushed with a warm saline solution every three hours and warm sweetened tea was given in a spoon frequently. In a few days recovery was complete.

